# Transformation of the Title V Maternal and Child Health Services Block Grant

**DOI:** 10.1007/s10995-015-1736-8

**Published:** 2015-02-18

**Authors:** Michael C. Lu, Cassie B. Lauver, Christopher Dykton, Michael D. Kogan, Michele H. Lawler, Lauren Raskin-Ramos, Kathy Watters, Lee A. Wilson

**Affiliations:** Maternal and Child Health Bureau, Health Resources and Services Administration, 5600 Fishers Lane, Rockville, MD 20857 USA

**Keywords:** Performance measurement, Health services, Transformation, Title V Block Grant

## Abstract

This paper describes the transformation of the Title V Maternal and Child Health (MCH) Services Block Grant. The Maternal and Child Health Bureau of the Health Resources and Services Administration led a 21-month visioning process to engage input from MCH stakeholders and other national, state and local MCH leaders, families and other partners to improve, innovate, and transform the Title V MCH Services Block Grant. The process has helped inform the development of a new grant guidance for the next 5-year cycle beginning in fiscal year 2016. The triple aims of the transformation are to reduce burden, maintain flexibility, and increase accountability. State reporting burden is reduced by aligning and streamlining the needs assessment, annual report and application, reducing the number of forms States have to fill out, eliminating Health Systems Capacity Indicators, and prepopulating the annual report and application with State data using national data sources. State flexibility is maintained through the needs assessment process whereby State needs and priorities drive the selection of National Performance Measures and State-specific Performance Measures, and the development of State Action Plan and Evidence-based/informed Strategy Measures. Accountability is increased through the new three-tiered performance measurement framework, which will help States tell a more coherent and compelling story about the impact of Title V on the health of the Nation’s mothers, children, and families. The ultimate success of the transformation will be measured by how much the transformed Title V program moves the needle in MCH in the States and for the Nation.

## Introduction

Enacted in 1935 as part of the Social Security Act, Title V is the oldest public health program in the Nation today [1, 2]. For more than three-quarters of a century, Title V has provided a foundation for ensuring the health and wellbeing of the Nation’s mothers, children and youth, including children and youth with special healthcare needs, and their families. Title V was converted to a Block Grant Program in 1981 [3].

Today the Title V Maternal and Child Health (MCH) Services Block Grant serves approximately 40 million people annually, including 2.3 million pregnant women, 4.1 million infants, 28.6 million children and 3.2 million children with special healthcare needs in 2014 [4]. Congress appropriated $634 million for the Block Grant in 2014, of which nearly $531 million were disbursed to 59 states and jurisdictions by formula, with the remainder allocated to Special Projects of Regional and National Significance (SPRANS) and Comprehensive Integrated Service System (CISS). With State matching fund, however, the combined federal-state funding for Title V Block Grant exceeds $6 billion annually [4], making it one of the largest public health programs for children and families in the nation.

Since March 2013, the Maternal and Child Health Bureau (MCHB), along with the Association of Maternal and Child Health Programs (AMCHP) and other national, state and local MCH leaders, stakeholders, families and other partners, have led a visioning process to improve, innovate and transform the Title V MCH Services Block Grant. This process has helped inform the development of the new grant guidance for the next 5-year cycle beginning in 2016. In this paper we describe the reasons for transformation, the visioning process, and how the new guidance and measurement system operationalize the triple aims of the transformation to reduce burden, maintain flexibility, and improve accountability.

## Why Transformation

From its inception, Title V’s mission has always been to improve the health and wellbeing of America’s children and families, but how it carries out its mission has changed over time in order to adapt to changing MCH population needs and environments [1]. Born out of the Great Depression, Title V provided grants to states to support MCH, child welfare, and “crippled children” in its early years [2]. In the 1960’s Title V authorized a number of new programs, including Maternity and Infant Project and Children and Youth Project, as part of the “War on Poverty.” With devolution of federal programs to state and local control beginning in the 1980’s, Title V was converted to a Block Grant program in 1981 by consolidating eight categorical programs. Nearly 85 percent of the funding went to the States, with very few measures of accountability for how the dollars were spent [2]. The Omnibus Budget Reconciliation Act of 1989 enacted stronger measures of accountability, and national performance measures were introduced in 1997, but states were held only loosely accountable for them.

The present transformation represents arguably the greatest transformation of the Title V program since it became a Block Grant in 1981. The need to transform is largely driven by rapid scientific advancements as well as changing health and political environments, which provide both unprecedented opportunities and threats. Scientific advancements over the past decade, especially in developmental origins of health and disease and life-course health development [5, 6], point to new opportunities to shift the curve on population health and human potential. Similarly, advancements in clinical care and public health have expanded our know-how for improving MCH. Expansion in healthcare coverage, first through Medicaid expansion for pregnant women, and then through the Children’s Health Insurance Program, and now with the Affordable Care Act (ACA), has improved access to a basic building block of health for millions of women, children, and families over the past two decades. The transformation is designed to help Title V capitalize on these growing opportunities for improving MCH.

These growing opportunities have coincided with major cutbacks in federal funding for Title V over the past decade. Since 2003, federal funding for the Block Grant has decreased by nearly $100 million. Expanded coverage under Medicaid and CHIP over the past two decades has shifted Title V’s role from a payer of last resort to the primary public health system for MCH populations in most states. With passage of the ACA, pressures mounted to reduce the Block Grant because improved coverage, it is argued, should lessen the need for the Block Grant as a payer of last resort. But because States do not routinely provide separate accounting of federal and state dollars and definitions of direct healthcare services vary across states, it remains unclear how much federal Block Grant dollars would be duplicated by expanded coverage under the ACA. Increasing budget pressures are also driving demand for greater accountability of performance and impact. But because States do many different things with their Title V dollars, it has been difficult to formulate a coherent and compelling national narrative about how Title V is moving the needle in MCH. Therefore, this transformation is designed to improve accountability of performance and impact, and better demonstrate the returns on investment for Title V in improving the health and wellbeing of the Nation’s mothers, children, and families.

## Methods

Beginning in March 2013, we have undertaken a visioning process to transform the Block Grant with a three-pronged approach. First, we convened internal workgroups at MCHB to take a fresh look at mission, vision, and values; performance measurement; and the Block Grant guidance and application, including needs assessment and the Block Grant review. Second, we asked Dr. Donna Peterson, Dean of the College of Public Health at the University of South Florida, to reach out to thought leaders in our field, not only supporters but also critics, to advise us on how we can improve, innovate and transform the Block Grant. Third, honoring our federal-state partnership, we asked AMCHP to convene a workgroup of its Board members to partner with us in the transformation process. Based on their initial recommendations, we developed a framework for transformation, and solicited input from the broader community of State Title V programs and other MCH leaders and stakeholders including families via a series of web-based “listening sessions,” which culminated in a town hall at AMCHP’s annual conference in February 2014. We also established a web-based drop box and received hundreds of emails from the field. We reached out to individuals and organizations representing important MCH stakeholders for their input, including family representatives to assure that family voices are heard, and that families are front and center in helping drive the transformation. All these inputs helped inform the development of the draft guidance and proposed measurement framework, which underwent two rounds of public comments beginning June 2014 as part of the Office of Budget and Management (OMB) approval process. The guidance was approved by the OMB in January 2015.

## Mission, Vision and Public Health Framework

While recognizing the need for transformation, this visioning process reminded us what is constant in Title V. It reaffirmed the mission of the Title V program to improve the health and well-being of the nation’s mothers, infants, children and youth, including children and youth with special healthcare needs, and their families [1]. It supported our vision of a Nation where all children and families are healthy and thriving, where every child and family have a fair shot at reaching their fullest potential.

And nothing speaks more clearly about the mission and vision of Title V than what it does for children and youth with special healthcare needs (CYSHCN). For more than three-quarters of a century, Title V worked to help CYSHCN reach their full potential by promoting early screening, diagnosis, and intervention, and assuring access to medical homes and community systems of care where the family is front and center in driving that care.

The visioning process also clarified Title V’s dual roles, both as a payer of last resort as well as a public health program. Even with the ACA, there will continue to be gaps in coverage and service for uninsured and underinsured pregnant women and children that the Title V Block Grant has to fill as payer of last resort. But Title V is first and foremost *the* public health system for MCH populations in all 59 states and jurisdictions, supporting States in carrying out the core public health functions of assessment, assurance and policy development, and the 10 essential services of public health [7]. In recent years, Title V has played a lead role in improving MCH outcomes in the States, including assuring universal newborn screening and timely follow-up, reducing infant mortality, and preventing child deaths and injuries. This transformation is designed to strengthen Title V’s role as the public health system for MCH populations, and the locus of accountability for improving the health of mothers, children, and families.

While reaffirming what is constant in Title V, the visioning process also sharpened our vision for transformation, with the triple aims of reducing burden, maintaining flexibility, and improving accountability.

## Reduce Burden

The first aim of the transformation is to reduce State reporting burden. We heard repeatedly from the States that the reporting requirements have become too burdensome, and so we set forth to reduce State reporting burden by half. We revised the guidance such that we would collect only the information we need and nothing more, and do so in a way that maximizes accountability and minimizes inefficiencies. Specifically, we realigned and streamlined the needs assessment, annual report and application to improve storytelling and reduce redundancy. We simplified, clarified, and reduced the number of forms that the States have to fill out from 21 to 11. We eliminated Health Systems Capacity indicators which have outlived their usefulness. Wherever possible, MCHB will prepopulate the annual report and application with state-specific data from national data sources, rather than asking the states to look for their own data. This will help reduce States’ data reporting burden, while improving data standardization across the Nation. Lastly, we completely revamped our Title V Information System (TVIS) to improve ease of use and minimize burden to the states.

## Maintain Flexibility

A second aim of the transformation is to maintain State flexibility. While recognizing the need for a more coherent and compelling national narrative about the impact of Title V, we believe strongly in the need to maintain flexibility for the States in how they use their Block Grant to address the unique needs and priorities of their MCH populations. That flexibility also strengthens Title V as a “co-laboratory” of 59 States and jurisdictions and thousands of local communities to drive improvements and innovations in MCH. But that flexibility does not make the Block Grant a blank check to the States. There needs to be a clear logic model in how the State proposes to use its Block Grant to drive improvements in MCH—how their 5-year Needs Assessment drives the selection of their 7–10 state priorities in MCH; how these priorities, in turn, inform the selection of the 8 of 15 national performance measures and 5 or more state-specific performance measures; and how these measures guide the development of the State Action Plan, complete with clear goals, SMART objectives and benchmarks, and evidence-based or evidence-informed strategies for each performance measure. States (and their local partners) are the driving force behind the needs assessment, selection of State priorities, national and state-specific performance measures, development of the State Action Plan and evidence-based/informed strategy measures, and ultimately improved outcomes for their MCH populations.

## Improve Accountability

A third aim of the transformation is to improve accountability. Accountability is about taking responsibility for one’s actions, measuring results, and delivering impact. Ultimately it is about accounting for how Title V is improving health and growing potential for the Nation’s mothers, children, and families. Programmatic accountability is improved by using a logic model to guide the development and integration of State needs, priorities, strategies, and measurement, and by revamping the annual report and application so they tell a more coherent story of Title V in the States. Financial accountability is improved by separating accounting of how federal dollars and State match are used, and by distinguishing reimbursable direct services from non-reimbursable primary and preventive services and public health services.

Most importantly, we improved accountability with the new three-tiered measurement framework described elsewhere in greater detail by Kogan et al. [8] in this issue. The first tier consists of National Outcome Measures (NOMs), which include population-level measures (many of which are legislatively mandated) of health outcomes which we expect Title V to improve over the next 5 years, as well as health status which we expect Title V to track irrespective whether the needle is movable by Title V (e.g. the rising prevalence of CYSHCN especially those with mental and behavioral health problems) given Title V’s core public health function of assessment. The second tier consists of performance measures, which include the list of 15 National Performance Measures (NPMs) from which the states will prioritize 8, based on their needs assessment and State priorities, as well as 5 or more state-specific performance measures (SPMs). We ask that the States select at least one measure from each of the six MCH domains (women’s and maternal health, perinatal and infant health, child health, adolescent health, children with special health care needs, and “life-course” or cross-cutting) to assure coverage across MCH life course. The third tier consists of Evidence-based/informed Strategy Measures (ESMs), which should follow from the evidence-based or evidence-informed strategies outlined in the State Action Plan. If public health is about having the right programs, policies, and systems in place to carry out the core functions of public health, then these ESMs are designed to hold Title V accountable for having the right programs, policies and systems in place to move the needle in MCH. An example of this 3-tiered measurement framework consists of infant mortality as a NOM, breastfeeding (to reduce SIDS and SUID) as a NPM, and the percent of infants born in a baby-friendly hospital in the State as a ESM, with the expectation that improvements in structures or processes will drive improvements in performance, which in turn will drive improvements in outcome.

## Strengthening Partnerships

Improving accountability goes both ways. States need to hold their federal partner (MCHB) accountable for helping them move the needle in MCH. In the past year, we supported the development of a new MCH Workforce Development Center to help retool the Title V MCH workforce in the States, sharpening their tools to drive improvements in access, quality, integration, equity, and accountability. We also established a network to support collaborative improvement and innovation (CoIIN) across States for reducing infant mortality and improving birth outcomes. Going forward, we will continue to look for opportunities to support State efforts to improve MCH by realigning SPRANS, CISS, and other MCHB investments with State Block Grant needs and priorities.

But we cannot improve MCH in our Nation by working in siloes. Title V must forge closer collaborative relationships with Title XIX (Medicaid) at the federal and state levels. We must also continue to strengthen our partnerships with the Administration on Children and Families, Centers for Disease Control and Prevention, and other health and human services programs. But recognizing that there are important social determinants, operating across the life course, that are the real drivers of MCH and health disparities, Title V must also continue to reach across sectoral boundaries and institutional siloes to partner with Departments of Agriculture, Education, Housing and Urban Development, and other programs, as well as national, state, community and family leaders in the private sector, in order to drive greater collective impact. That is what it will take for Title V to be *the* locus of accountability for improving the health of the Nation’s mothers, children and families.

In summary, through a 21-month visioning process with input from diverse MCH stakeholders and other national, state and local MCH leaders, families and other partners, the Title V MCH Services Block Grant has been transformed. This transformation has improved the ability of States to tell a more coherent and compelling narrative about the impact of Title V, while reducing reporting burden and maintaining flexibility for the States. The ultimate success of the transformation will be measured by how much the transformed Title V program moves the needle in MCH in the States and for the Nation.
